# Supplementary material for: Molecular landscape of the fungal plasma membrane and implications for antifungal action
Source: Nat Commun. 2025 Oct 14;16:9125. doi: 10.1038/s41467-025-64171-x (PMC12521646; doi:10.1038/s41467-025-64171-x)
Supplement: Supplementary file 1 — Supplementary Information [file 41467_2025_64171_MOESM1_ESM.pdf]

**Supplementary Information**

**Supplementary Table 1.** The most abundant membrane-associated proteins identified from the proteomics analysis of *Candida glabrata* crude membranes.

| Protein Name | Description | MaxLFQ | Molecular Weight (Da) | Oligomerization |
|---|---|---|---|---|
| **Pst2** | quinone oxidoreductase, eisosome-binding | 1.72E+09 | 20,975 | tetramer (5MP4) [1] |
| **Lsp1** | eisosome | 3.95E+08 | 35,056 | dimer (3PLT) [2,3] |
| **Pst3** | quinone oxidoreductase, eisosome-binding | 2.91E+08 | 29,747 | unknown |
| **Ugp1** | UDP-glucose pyrophosphorylase | 2.67E+08 | 55,951 | octamer[4] |
| **Pil1** | eisosome | 2.62E+08 | 35,150 | dimer[2,3] |
| **Tpi1** | triosephosphate isomerase | 2.06E+08 | 26,870 | dimer (1NEY)[5] |
| **Met6** | methionine synthase | 2.02E+08 | 85,862 | monomer (4L64)[6] |
| **Fhn1** | eisosome | 7.35E+07 | 19,019 | unknown |
| **Hxt6/7** | high-affinity glucose transporter | 6.61E+07 | 61,546 | monomer |
| **Sfh5** | phosphatidylinositol transfer protein | 5.14E+07 | 34,685 | monomer (6W32)[7] |
| **Rfs1** | eisosome-binding | 4.88E+07 | 21,147 | unknown |
| **Pma1** | $H^+$-ATPase | 4.59E+07 | 98,376 | hexamer (7VH5)[8] |

| | | | | |
|---|---|---|---|---|
| **Fks1** | β-1,3-glucan synthase (GS) | 4.17E+07 | 213,914 | monomer (7XE4)[9] |
| **Sec4** | GTPase, vesicle-mediated exocytosis | 3.90E+07 | 23,681 | monomer (3CPH)[10] |
| **Nce102** | eisosome | 3.85E+07 | 19,433 | unknown |
| **Rho1** | GTPase, regulatory subunit of GS | 3.24E+07 | 23,160 | monomer (6JIK) |
| **Yck1** | casein kinase | 3.05E+07 | 61,538 | unknown |
| **Ptr2** | peptide transporter | 2.96E+07 | 67,846 | unknown |
| **Gas1** | 1,3-beta-glucanosyltransferase | 2.56E+07 | 59,940 | unknown |
| **Ykt6** | v-SNARE, vesicular trafficking | 2.31E+07 | 23,179 | unknown |
| **Gas2** | 1,3-beta-glucanosyltransferase | 2.23E+07 | 60,396 | monomer (5O9Q)[11] |
| **Aqy1** | aquaporin-1 | 2.20E+07 | 31,681 | tetramer (3ZOJ)[12] |
| **Ycp4** | quinone oxidoreductase, eisosome-binding | 2.01E+07 | 28,413 | unknown |
| **Fet3** | multicopper oxidase | 1.91E+07 | 72,020 | monomer (1ZPU)[13] |
| **Mrh1** | unknown | 1.84E+07 | 35,107 | unknown |
| **Hxt4** | high-affinity glucose transporter | 1.75E+07 | 62,918 | monomer |
| **Sec1** | exocytosis | 1.60E+07 | 80,753 | unknown |
| **Pst1** | cell wall integrity | 1.46E+07 | 44,050 | unknown |

| | | | | |
|---|---|---|---|---|
| **Fat1** | long-chain fatty acid transporter | 1.41E+07 | 77,847 | unknown |
| **Osh2** | lipid transport protein | 1.39E+07 | 142,521 | unknown |
| **End3** | endocytosis | 1.32E+07 | 39,884 | unknown |
| **Inp53** | endocytosis | 1.31E+07 | 124,185 | unknown |
| **Fks2** | isoform of β-1,3-glucan synthase (GS) | 1.29E+07 | 217,622 | unknown |
| **Ras1** | GTPase | 1.25E+07 | 37,053 | monomer (7NZZ)[14] |
| **Ssy5** | endopeptidase | 1.16E+07 | 76,231 | unknown |
| **Kre6** | glucosyl hydrolase, cell wall synthesis | 1.13E+07 | 77,588 | unknown |

**Supplementary Table 2.** Quantitative evaluation of convolutional neural network (CNN)-based annotation using manual annotation as a benchmark.

| | manual annotation | CNN-based annotation | | true positive | false negative | false positive | F1 score |
|---|---|---|---|---|---|---|---|
| | | threshold | total picked | | | | |
| ribosome | 177 | 0.45 | 265 | 172 | 5 | 88 | 0.788 |
| | | 0.50 | 263 | 171 | 6 | 86 | 0.788 |
| | | 0.55 | 260 | 171 | 6 | 83 | 0.793 |
| Pma1 | 451 | 0.45 | 669 | 222 | 229 | 218 | 0.498 |
| | | 0.50 | 607 | 194 | 257 | 156 | 0.484 |
| | | 0.55 | 560 | 159 | 292 | 109 | 0.443 |

**Supplementary Table 3.** Analysis of Pma1 distribution in plasma membrane tomograms collected from different samples as determined by manual labeling, Gaussian Mixture Model (GMM) and *k*-means clustering methods.

| sample (–/+ CSF) | number of tomograms | number of Pma1 | Number of Pma1 clusters | | |
|---|---|---|---|---|---|
| | | | manual annotation | GMM clustering analysis | *k*-means clustering analysis |
| CBS138 (–) | 14 | 876 | 71 | 71 | 71 |
| CBS138 (+) | 16 | 1469 | 52 | 50 | 52 |
| KH238 (–) | 19 | 3289 | 155 | 153 | 155 |
| KH238 (+) | 7 | 455 | 24 | 24 | 24 |

**Supplementary Table 4. Statistical analysis of Pma1 clustering across various samples.** The Kruskal-Wallis H test yielded $p < 0.005$ for both average cluster radius and intra-cluster distance. Post-hoc multiple comparisons were performed using Dunn's test. S1 = CBS138 without CSF; S2 = CBS138 with CSF; S3 = KH238 without CSF; S4 = KH238 with CSF.

| clustering metric | Kruskal-Wallis test | | estimated power | Post-hoc (Dunn's) *p*-value | | | | | |
|---|---|---|---|---|---|---|---|---|---|
| | H test value | *p*-value | | S1-S2 | S1-S3 | S1-S4 | S2-S3 | S2-S4 | S3-S4 |
| average cluster radius | 19.803 | 0.0002 | 0.8967 | 0.0036 | 0.586 | 0.0027 | 0.0011 | 0.8561 | 0.0008 |
| average intra-cluster distance | 18.3275 | 0.0004 | 0.9266 | 0.0051 | 0.717 | 0.0023 | 0.0029 | 0.9903 | 0.0014 |

a

| Strain | CSF concentration (μg/ml) |
|---|---|
| Parent (CBS138) | 0.25 |
| YFP-Fks1 (IGCg1) | 0.25 |
| Fks1-OE (KH238) | 0.25-0.5 |
| Δfen1 (MVKCg9) | 1.5 |

b

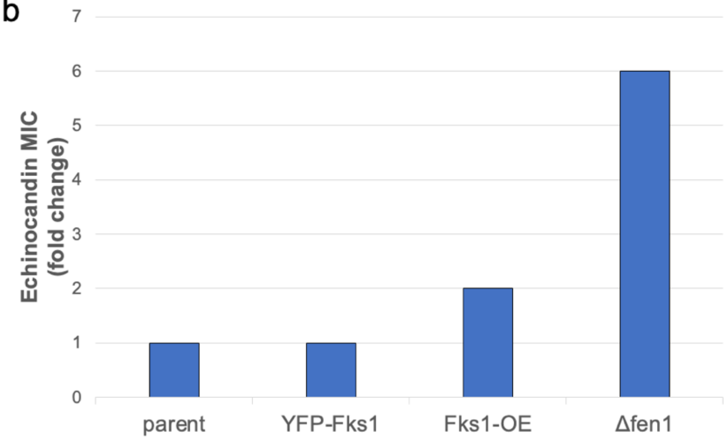


**Supplementary Figure 1. Antifungal susceptibility testing of various *C. glabrata* strains.** (**a**) Caspofungin (CSF) minimum inhibitory concentration (MIC) ranges for various *C. glabrata* strains used in this study. The IGCg1 strain expresses YFP-Fks1. The KH238 strain overexpresses Fks1. The MVKCg9 strain harbors a deletion of *FEN1,* which encodes a fatty acid elongase. (**b**) CSF susceptibility results expressed as fold changes in the MIC value relative to the parent strain.

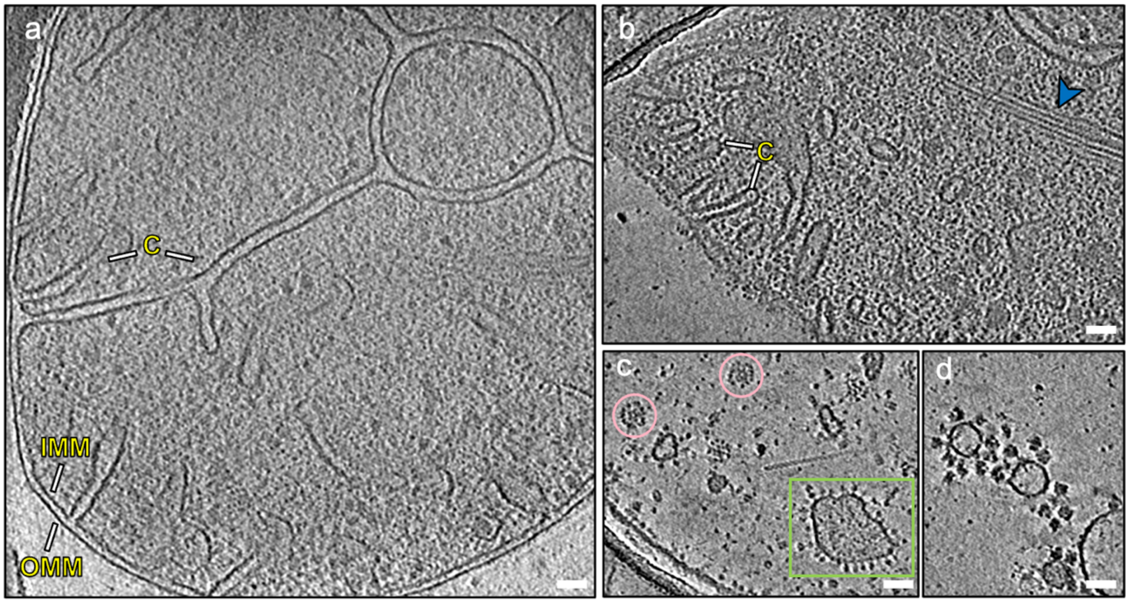


**Supplementary Figure 2. Visualization of diverse cellular organelle features in *C. glabrata* crude membrane preparations.** Slice views of cellular tomograms depicting various subcellular features: (**a**) intact mitochondrion with distinct outer membrane (OMM), inner membrane (IMM) and cristae (C), (**b**) mitochondrial membrane with distinct inner membrane cristae (C) and filament bundles (blue arrowhead), (**c**) pyruvate dehydrogenase complexes (pink circles) and ATP synthases decorating the mitochondrial membrane (green box), and (**d**) membrane-associated ribosomes. Scale bars, 50 nm.

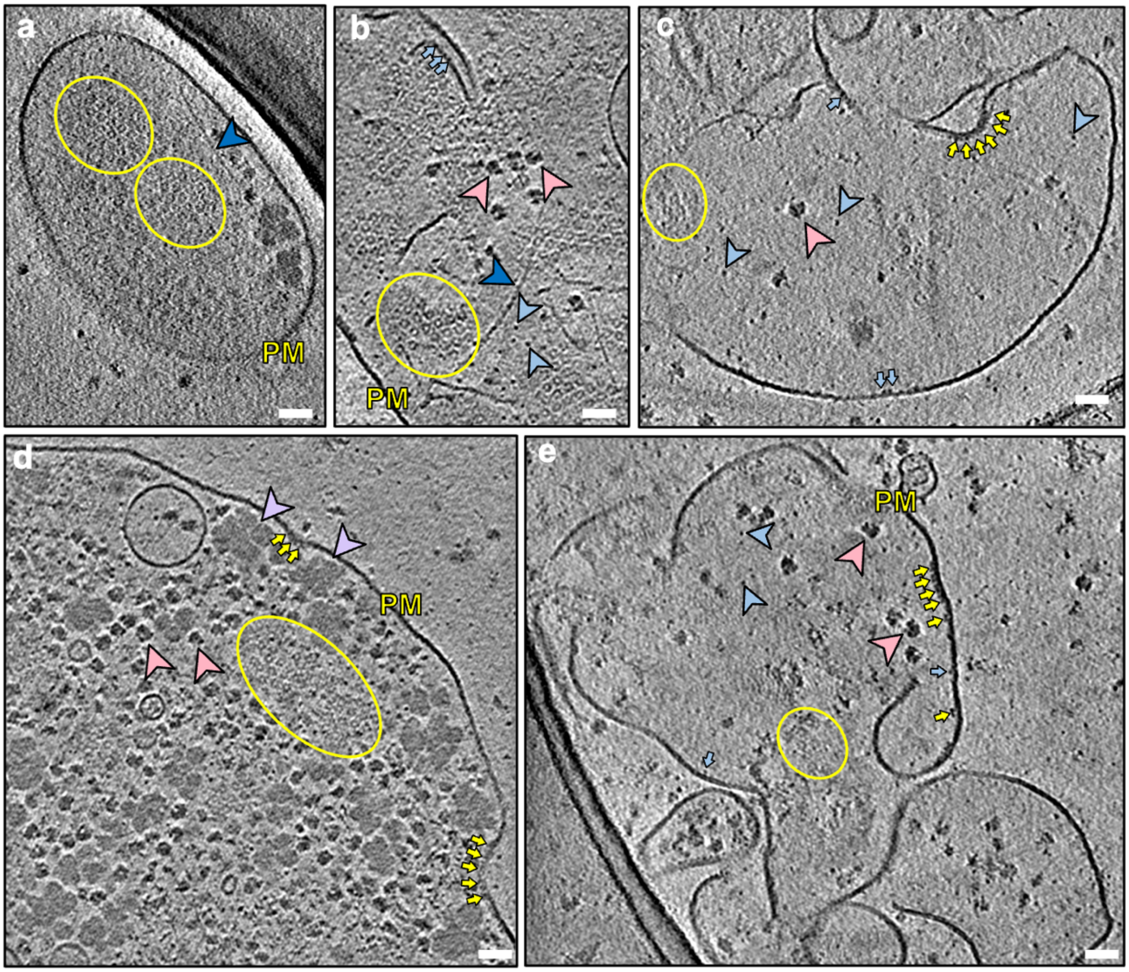


**Supplementary Figure 3. Cryo-ET reveals diverse cellular structures and macromolecular complexes in crude membrane preparations. (a-e)** Slice views of tomograms of crude membrane preparation depicting various structural features including the plasma membrane (PM), actin filament (dark blue arrowheads in a and b), ribosomes (pink arrowheads in b-e), clusters of Pma1 hexamers (yellow circle, a-e), side views of Pma1 hexamers (yellow arrows in c-e), glycogen storage granules (purple arrowheads in d), and glucan synthase complex (light blue arrowheads; side views of putative GS are denoted by light blue arrows in b, c, and e). Scale bars, 50 nm.

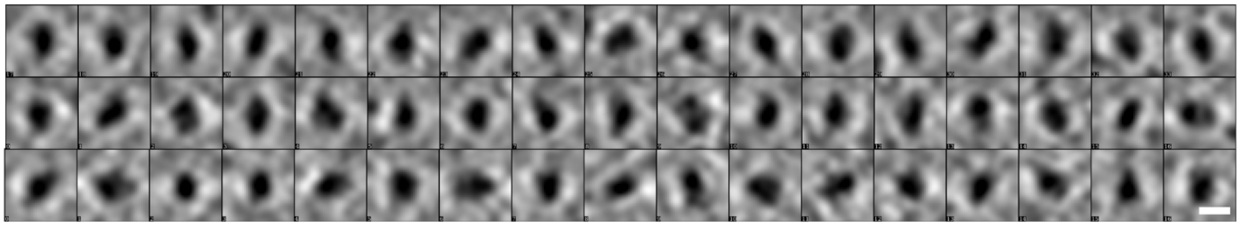

**Supplementary Figure 4. 2D slice views of GS subtomograms selected from plasma membrane tomograms.** Scale bar, 10 nm.

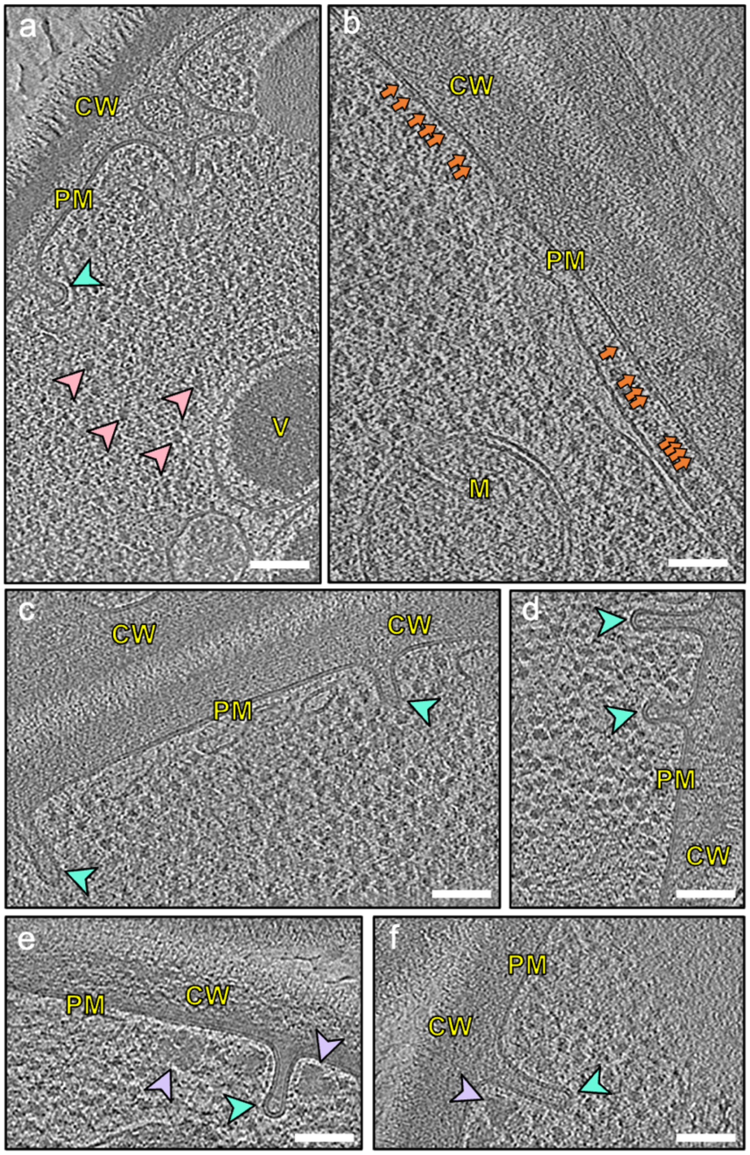


**Supplementary Figure 5. Cryo-ET of lamellae of *C. glabrata* cells reveals the fungal plasma membrane microdomain organization.** Cellular structures are clearly visible: cell wall (CW), plasma membrane (PM), eisosome (teal arrowheads), ribosome (pink arrowheads), vacuole (V), plasma membrane protein complexes (orange arrows), mitochondrion (M), glycogen granule (purple arrowheads). Scale bars, 100 nm.

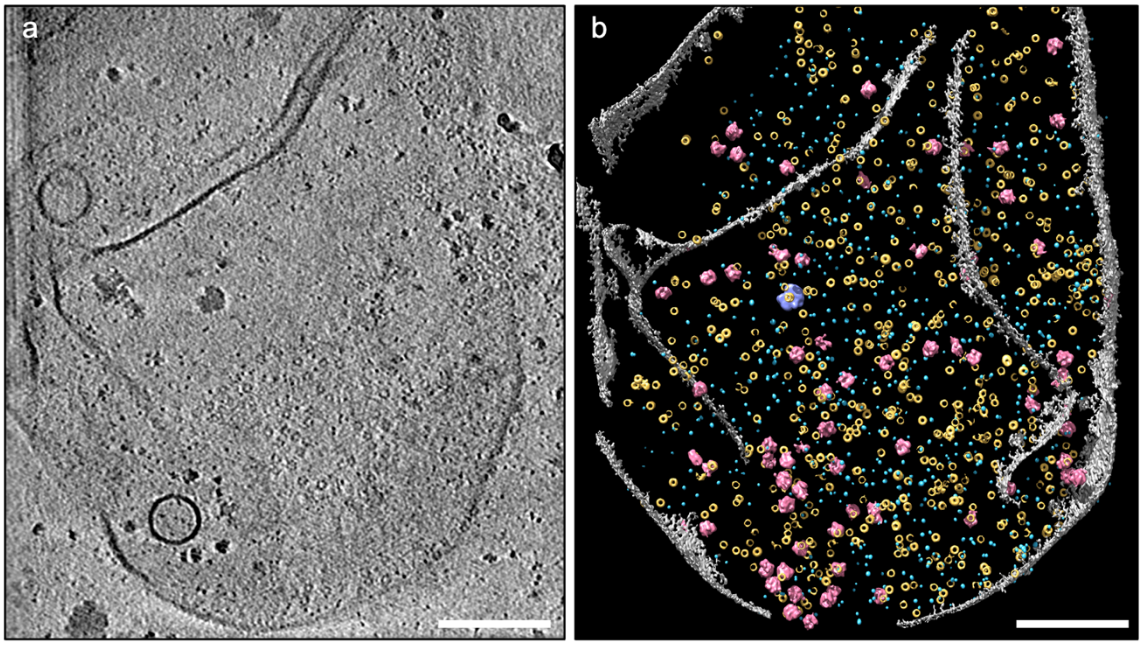


**Supplementary Figure 6. Annotation of a tomogram of plasma membranes generated from CSF-treated wild type spheroplasts.** (**a**) Slice view of the tomogram showing the distribution pattern of various molecular species following CSF exposure. This tomogram is representative of a dataset comprising over 100 tomograms acquired from more than three independent samples. (**b**) Annotation and 3D visualization of the tomogram in (a), depicting the plasma membrane (gray), Pma1 hexamers (yellow), GS densities (light blue), ribosomes (pink), and glycogen granules (purple). Scale bars, 200 nm.

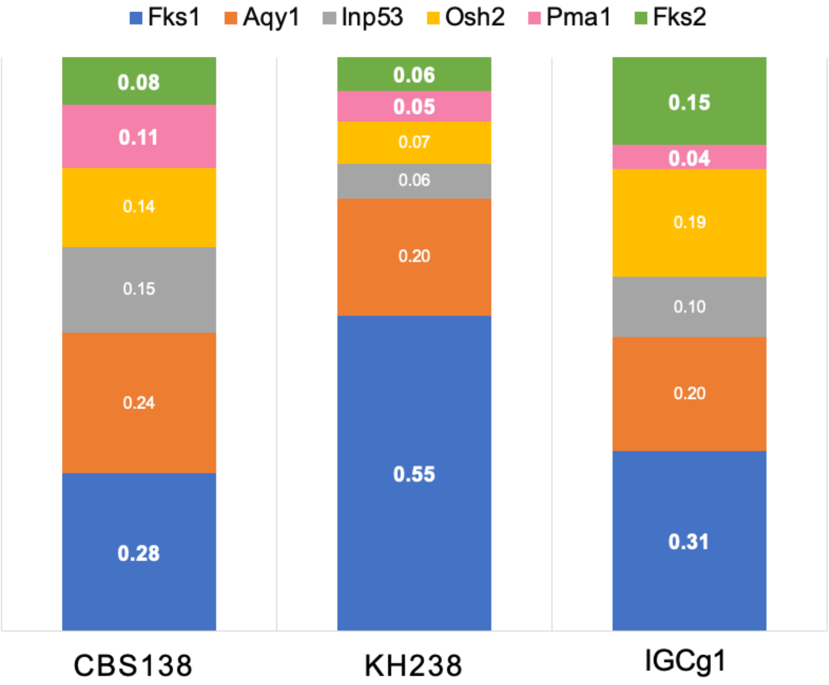


**Supplementary Figure 7. Quantitative mass spectrometry analysis of large (>100 kDa), abundant integral membrane protein complexes in crude membrane preparations.** Crude membrane fractions were isolated from CBS138 (wild type), KH238 (Fks1-overexpressing), and IGCg1 (YFP-Fks1) strains. The total abundance of the six selected protein complexes was normalized across the three samples. The relative abundance of each complex is shown as a percentage of the total abundance of the six proteins. Source data are provided as a Source Data file.

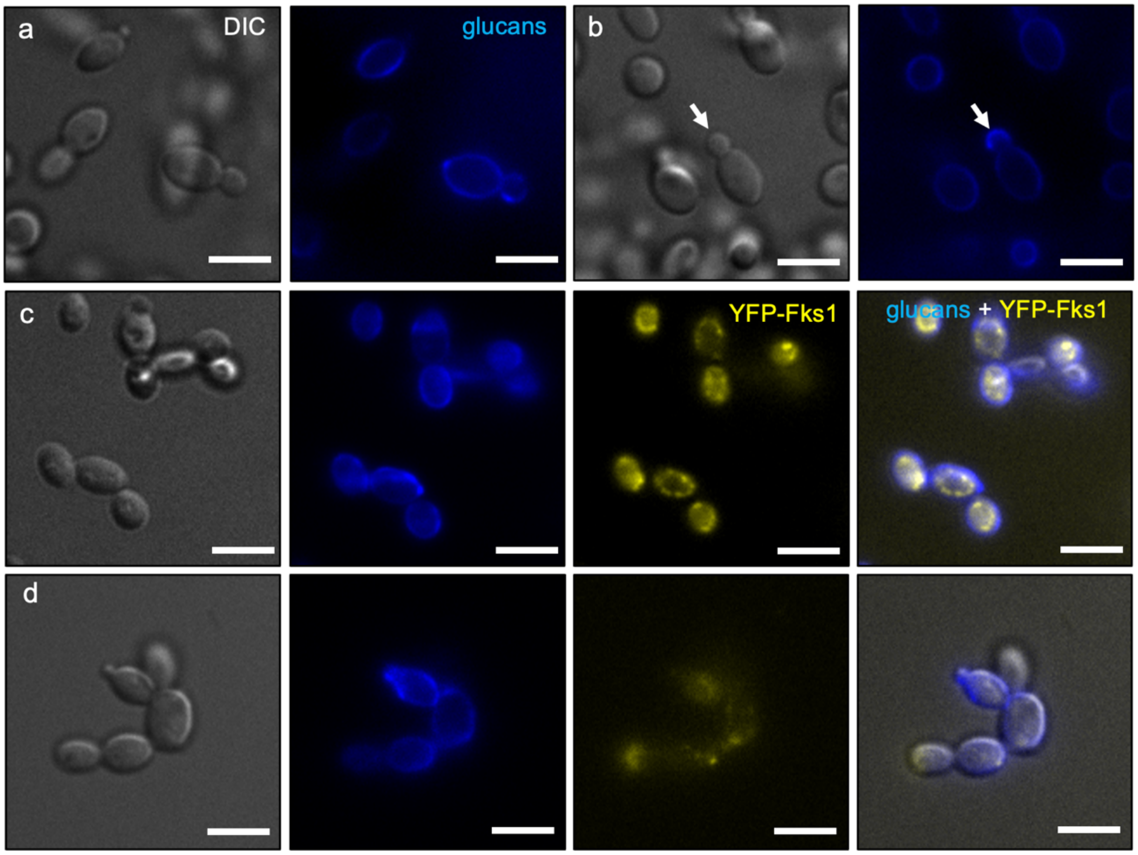


**Supplementary Figure 8. Caspofungin (CSF) treatment altered the distribution of glucan synthase (GS) within *C. glabrata* cells.** (**a-b**) CBS138 (wild type) strain stained with glucan-specific aniline blue to reveal the *in vivo* glucan levels within the cell wall. White arrows indicate nascent budding cells. (**c-d**) IGCg1 (YFP-Fks1) strain stained with aniline blue. YFP signals indicate the subcellular localization of GS within untreated (c) and CSF-treated (d) cells. Scale bars, 5 μm.

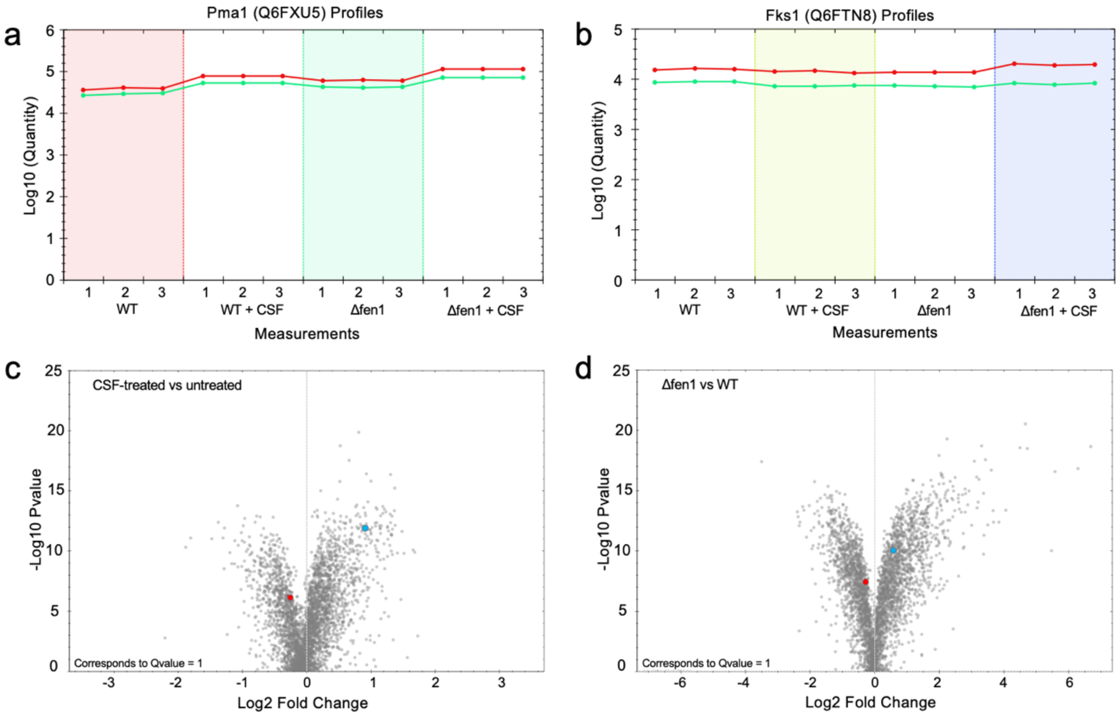


**Supplementary Figure 9: Caspofungin (CSF) treatment does not significantly alter the relative abundance of Fks1 and Pma1 complexes in wild type or Δfen1 mutant cells. (a, b)** Quantitative mass spectrometry analysis of Pma1 (a) and Fks1 (b) levels in crude membrane preparations from untreated wild type, CSF-treated wild type, untreated Δfen1 mutant, and CSF-treated Δfen1 mutant cells. The results represent technical triplicates. Red lines denote MS1, precursor ion mass-to-charge ratios; Green lines denote MS2, fragment ion spectra. **(c, d)** Volcano plots showing protein abundance changes in crude membrane preparations from CSF-treated versus untreated wild type cells (c), and Δfen1 mutant versus wild type cells (d). In both plots, Pma1 is marked in blue and Fks1 in red. The increase in Pma1 levels in CSF-treated samples in (c) may reflect the loss of loosely associated membrane protein complexes after CSF treatment, resulting in a relative enrichment of Pma1

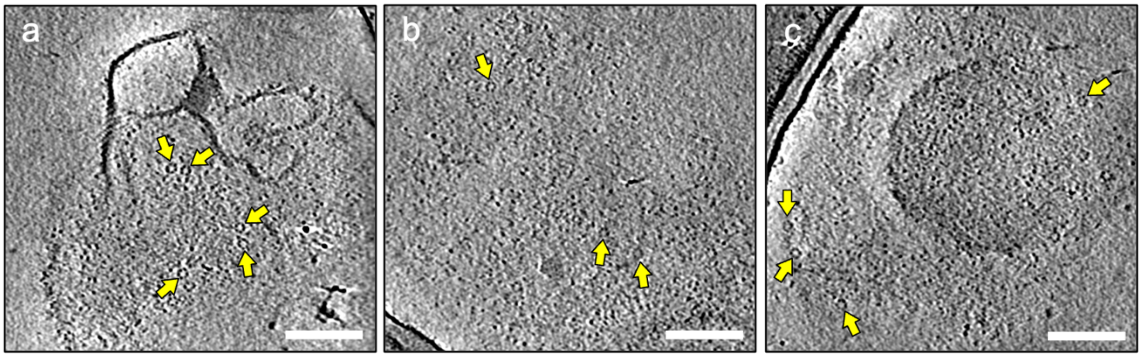


**Supplementary Figure 10. Cryo-ET of plasma membranes from the Δfen1 mutant shows compromised membrane structure and dispersed Pma1 complexes.** (**a-c**) Slice views of tomograms of plasma membranes from Δfen1 mutant cells. Loose Pma1 hexamers are indicated by yellow arrows. Scale bars, 100 nm. This tomogram is representative of a dataset comprising over 20 tomograms.

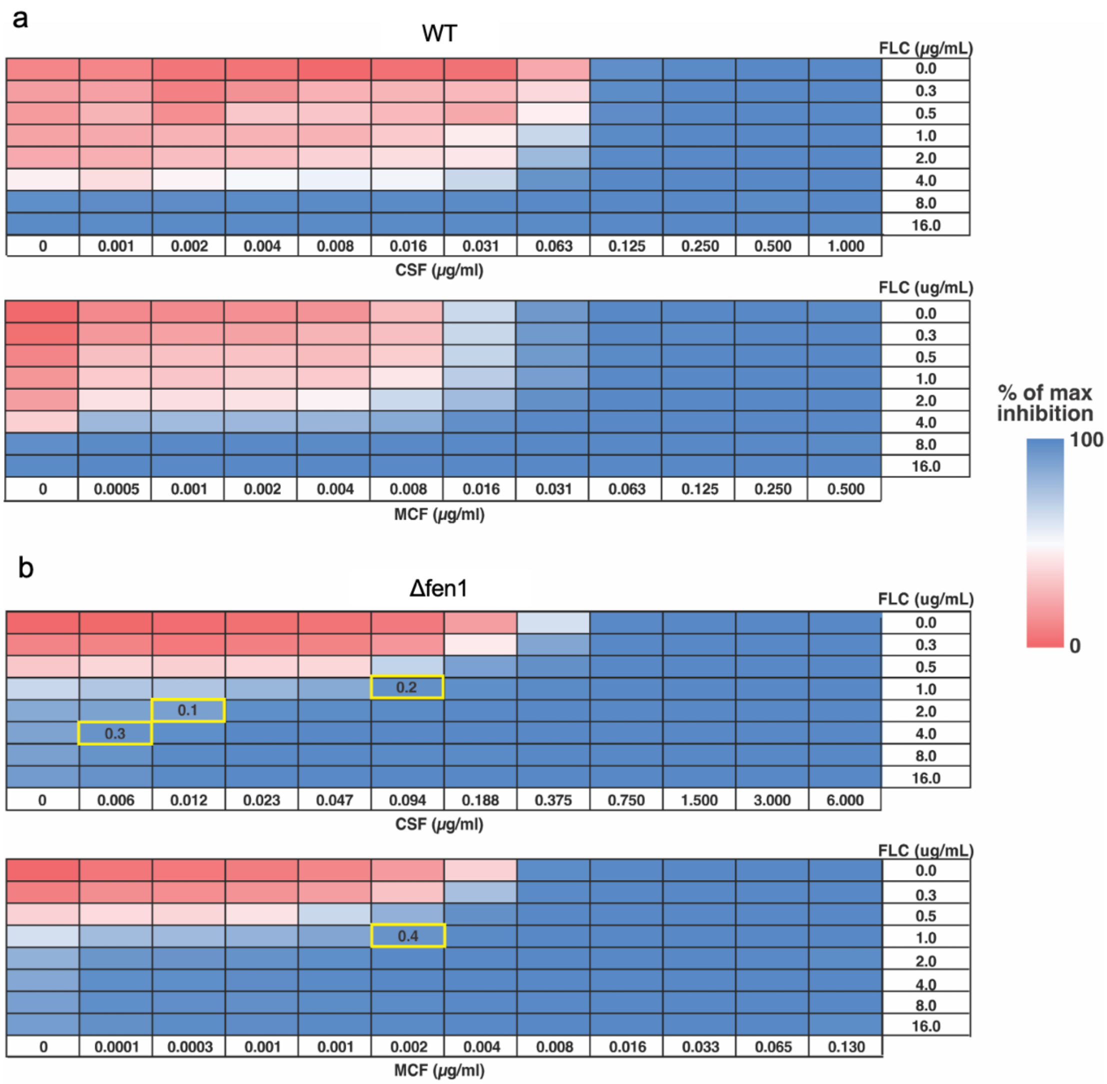


**Supplementary Figure 11. Checkerboard assays examining the impact of ergosterol synthesis inhibition on echinocandin susceptibility in wild type (a) and Δfen1 mutant (b) strains.** Combined fluconazole (FLC) with either caspofungin (CSF) or micafungin (MCF) revealed no significant drug interaction in wild type cells, and a minor interaction in the Δfen1 mutant. Yellow boxes: wells with synergistic interactions with Fractional inhibitory concentration indices (FICI) values inside. Source data are provided as a Source Data file.

## Supplementary References

1 Koch, K. *et al.* Structure, biochemical and kinetic properties of recombinant Pst2p from Saccharomyces cerevisiae, a FMN-dependent NAD(P)H:quinone oxidoreductase. *Biochim Biophys Acta Proteins Proteom* **1865**, 1046-1056 (2017). https://doi.org/10.1016/j.bbapap.2017.05.005

2 Karotki, L. *et al.* Eisosome proteins assemble into a membrane scaffold. *J. Cell Biol.* **195**, 889-902 (2011). https://doi.org/10.1083/jcb.201104040

3 Ziolkowska, N. E., Karotki, L., Rehman, M., Huiskonen, J. T. & Walther, T. C. Eisosome-driven plasma membrane organization is mediated by BAR domains. *Nat. Struct. Mol. Biol.* **18**, 854-856 (2011). https://doi.org/10.1038/nsmb.2080

4 Roeben, A. *et al.* Structural basis for subunit assembly in UDP-glucose pyrophosphorylase from Saccharomyces cerevisiae. *J. Mol. Biol.* **364**, 551-560 (2006). https://doi.org/10.1016/j.jmb.2006.08.079

5 Jogl, G., Rozovsky, S., McDermott, A. E. & Tong, L. Optimal alignment for enzymatic proton transfer: structure of the Michaelis complex of triosephosphate isomerase at 1.2-A resolution. *Proc Natl Acad Sci U S A* **100**, 50-55 (2003). https://doi.org/10.1073/pnas.0233793100

6 Ubhi, D., Kago, G., Monzingo, A. F. & Robertus, J. D. Structural analysis of a fungal methionine synthase with substrates and inhibitors. *J. Mol. Biol.* **426**, 1839-1847 (2014). https://doi.org/10.1016/j.jmb.2014.02.006

7 Khan, D. *et al.* A Sec14-like phosphatidylinositol transfer protein paralog defines a novel class of heme-binding proteins. *Elife* **9** (2020). https://doi.org/10.7554/eLife.57081

8 Zhao, P. *et al.* Structure and activation mechanism of the hexameric plasma membrane H+-ATPase. *Nature Communications* **12**, 6439 (2021). https://doi.org/10.1038/s41467-021-26782-y

9 Hu, X. *et al.* Structural and mechanistic insights into fungal β-1,3-glucan synthase FKS1. *Nature* (2023). https://doi.org/10.1038/s41586-023-05856-5

10 Ignatev, A., Kravchenko, S., Rak, A., Goody, R. S. & Pylypenko, O. A structural model of the GDP dissociation inhibitor rab membrane extraction mechanism. *J. Biol. Chem.* **283**, 18377-18384 (2008). https://doi.org/10.1074/jbc.M709718200

11 Delso, I. *et al.* Inhibitors against Fungal Cell Wall Remodeling Enzymes. *ChemMedChem* **13**, 128-132 (2018). https://doi.org/10.1002/cmdc.201700720

12 Fischer, G. *et al.* Crystal structure of a yeast aquaporin at 1.15 angstrom reveals a novel gating mechanism. *PLoS Biol.* **7**, e1000130 (2009). https://doi.org/10.1371/journal.pbio.1000130

13 Taylor, A. B., Stoj, C. S., Ziegler, L., Kosman, D. J. & Hart, P. J. The copper-iron connection in biology: structure of the metallo-oxidase Fet3p. *Proc Natl Acad Sci U S A* **102**, 15459-15464 (2005). https://doi.org/10.1073/pnas.0506227102

14 Manso, J. A. *et al.* Pathogen-specific structural features of Candida albicans Ras1 activation complex: uncovering new antifungal drug targets. *mBio* **14**, e0063823 (2023). https://doi.org/10.1128/mbio.00638-23
