# Supplementary material for: Molecular landscape of the fungal plasma membrane and implications for antifungal action
Source: Nat Commun. 2025 Oct 14;16:9125. doi: 10.1038/s41467-025-64171-x (PMC12521646; doi:10.1038/s41467-025-64171-x)
Supplement: Supplementary file 2 — Description of Additional Supplementary Files [file 41467_2025_64171_MOESM2_ESM.pdf]

Description of Additional Supplementary Files:

Supplementary Movie 1: Slice views and annotation of a *C. glabrata* wild type plasma membrane tomogram showing the molecular landscape of the fungal plasma membrane.

Supplementary Movie 2: Slice views of a *C. glabrata* cryo-lamella tomogram revealing the cell wall, plasma membrane microdomain organization, and subcellular features.

Supplementary Movie 3: Micropipette aspiration of untreated *C. glabrata* wild type spheroplasts.

Supplementary Movie 4: Micropipette aspiration of caspofungin-treated *C. glabrata* wild type spheroplasts.

Supplementary Movie 5: Slice views and tomogram annotation of plasma membranes generated from *C. glabrata* wild type spheroplasts treated with caspofungin.
